# Supplementary material for: Genome-wide binding analysis of 195 DNA binding proteins reveals “reservoir” promoters and human specific SVA-repeat family regulation
Source: PLoS One. 2021 Jun 24;16(6):e0237055. doi: 10.1371/journal.pone.0237055 (PMC8224974; doi:10.1371/journal.pone.0237055)
Supplement: Attachment — Submitted filename: response_to_reviewer_comments.docx [file pone.0237055.s012.docx]

5. Review Comments to the Author

Please use the space provided to explain your answers to the questions above. You may also include additional comments for the author, including concerns about dual publication, research ethics, or publication ethics. (Please upload your review as an attachment if it exceeds 20,000 characters)

Reviewer #1: In this paper Shehata et al present a very detailed re-analysis of DNA binding profiles of more than 190 DNA binding proteins in K562 cell lines, as derived from the ENCODE project. The authors present several confirmatory findings, which corroborate the results of previous studies, but also some original and new interesting observations. The structure of the paper is very rational and the English is good. The manuscript reads very well good. All in all, my evaluation of the work by Shetata et al is positive in general, however I some concerns/issues that the authors should address before the publication of their work:

We are very grateful for the reviewer’s encouragement and constructive criticisms that we will address below.

1) Since the authors make a point about the reproducibility of bioinformatics analyses, I believe that apart from sharing the code that was used to execute all their analyses, they should also make available some intermediate files and results. Including for example bigWig files with detailed coverage profiles, and/or Peak calling files in bed format. This could be easily done by building a track hub on the UCSC genome browser.

We agree with the reviewer, to this end we have now made a track hub on UCSC that has contains the bigWig files for all datasets in the study. We also added our individual peak files (MACS 2.0 broadPeak) and our consensus peak files (requiring peaks to overlap in both replicates). This is now available at: http://genome.ucsc.edu/cgi-bin/hgTracks?db=hg38&hubUrl=https://bchm5631sp2020.s3.amazonaws.com/trackhub/hub.txt

2) Related to 1, since also the ENCODE project has defined a very detailed and reproducible workflow for the analysis of their data (see https://www.encodeproject.org/pipelines/ENCPL138KID/), I believe that the authors could/should present a more detailed comparison of their results with those obtained/available from ENCODE. What are the main differences between the two pipelines? How many peaks are in common? How many are new? Is any systematic/consistent pattern observed?

The reviewer raises an important but complex point. For the samples we re-analyzed, ENCODE used their version 2.0 pipeline (https://www.encodeproject.org/pipelines/ENCPL367MAS/ ) which is very similar to the nf-core/chipseq pipeline in terms of peak calling and QC steps. It differs most in that it uses a different read aligner (bowtie2 vs. BWA). Since it is common practice to produce head-to-head comparisons each time a new software is released (for ex. <https://www.nature.com/articles/s41598-020-64655-4>) and there have been studies that comprehensively compare results using different alignment algorithms (<https://bmcbioinformatics.biomedcentral.com/articles/10.1186/1471-2105-14-184>), we feel that it would be out of the scope of this study to do so. Particularly when we reason that the differences would be driven primarily by the choice of aligner.

That said, overall, the nf-core/chipseq pipeline produced similar individual peak files and our consensus overlap is consistent with ENCODE’s “merged-replicate” peak files. In terms of finding reproducible peaks for each replicated dataset, 97% of the data was consistent with the output of the ENCODE pipeline (excluding the issue of tagged proteins – see below). Yet, as the reviewer insightfully suggests there are some differences:

i) Six ChIP experiments did not have any overlapping peaks in our pipeline, whereas ENCODE did find overlapping peaks in these six ChIP samples. Most concerning to us was the widely studied DNMT1 ChIP. Based on the reviewer’s suggestion we went back to further investigate the differences. What we found is that in this case ENCODE ran MACS2 with narrowPeak calls whereas nf-core used broadPeak calls (See figure below).

This resulted in many more peaks in each replicate. In turn, due higher probability of overlap with more peaks, overlapping peaks were found (Figure 1). Our study only used broadPeaks for consistency and MACS2 run with the broadPeaks setting does not find overlapping peaks across replicates.

Figure 1: Difference between narrowP eak and broadPeak “consensus peaks”. Individual broadPeaks are shown in light blue, narrowPeaks in blue and broadPeak merged blue box with arrows. Our pipeline using broadPeaks found a peak in replicate 2 but not replicate 1 (top). MACS2 narrowPeak calls have many more peaks and yet very little overlap across these peaks. It is encouraging that one of the narrowPeak calls in ENCODE overlaps with the single broadPeak from our pipeline. However, owing to the numerous peaks (thus more likely to overlap by chance) we took a conservative approach of using broadPeaks to reduce potential overlap due to “noisy” narrowPeak calls.

Considering how much of the data is consistent with ENCODE’s rigor and reproducibility we do not feel comfortable pointing out the small number of samples of data that deviate from the vast majority of ENCODE data. Or in other words the resource is so complete and reproducible we don’t want to discredit the few samples that deviate. Nonetheless, if the reviewer finds this an important aspect to note, we will include our example detailed analysis of DNMT1 and note the other five samples.

ii) Another concerning aspect that we observed was the similarity of peak profiles for eGFP tagged proteins (Figure 2C). The similarity of these ChIP datasets is concerning, considering the diversity of protein functions represented in these tagged samples. However, we could not find any in-depth analysis performed by ENCODE on these samples. We note this concern in the text as a precaution for future users of these ENCODE datasets.

3) While the code as available from their main Github repository is very tidy and well commented, I believe that authors should also write a "proper" material and methods section. For example, by reading the paper it is not exactly clear which assembly of the genome assembly they used in their analysis, and/or also the reference annotation of the genome that was used. More details on the tools and software libraries that were used should also be provided. A scientific paper is usually intended to have a broad scientific audience. The methods should be explained clearly, and be intelligible to everyone, not only to bioinformaticians or people that are proficient in the R programming language.

We agree, and thank the reviewer. We are embarrassed that we did not include this. We have now added a proper methods section describing data sources, versions of pipelines and other needed information such as statistical approaches and annotation files. We further made sections for each data type and included there the considerations needed in order to reproduce this study.

4) Sometimes authors introduce hard cut-off for the binning of the data. See for example, gene expression data: Low: (0.001,0.137] TPM, how were these cut-offs derived? More details should be provided. To be honest I did not understand completely which type of RNA-seq data were used for the estimation of gene expression levels, but also how the data were processed. Were these obtained directly from ENCODE? Please specify

We are glad the reviewer brought this up. We taught the students to not make “subjective cutoffs” e.g., the classic 2-fold etc. Rather we had them use “quantile-based” cutoffs. Thus, the cutoffs of 0.137 and 3 correspond roughly to the 50^th^ and 75^th^ percentile of expression respectively. Since there are so many unexpressed genes, the 25^th^ percentile corresponded to a TPM of 0. In order to differentiate between genes with very low expression and those that have no reads, we added the cutoff of 0.01. While we note that there is no perfect way to make cutoffs in a continuous distribution (RNA-seq reads are typically modeled with a negative binomial distribution), it is important to have a rationale and we tried to demonstrate the importance of that to the class by using quantile-based cutoffs. We have now clarified this in the methods and main text and added the quantile and rationale for these cutoffs. The reviewer’s point is also taken and corrected for other sections using distribution-based cutoffs.

Additionally, we’ve now included a clarification on the source of the RNA-seq counts in the main text and in the new methods section. In short, in order to ensure that the quantification matched our annotation Gencode v32, the aligned BAM files were retrieved from ENCODE and those were quantified using Rsubread featureCounts.

5) The authors should elaborate a little on the concept of "reservoir promoter". This is not explained very clearly in the introduction. And while I can grasp the concept, I would like to see a more detailed explanation of possible molecular mechanisms/implications. While I believe that this is a reasonable and fascinating hypothesis, currently it is not backed up by experimental evidence. So, several sentences in the paper regarding the existence and possible functional roles of reservoir promoters should be toned down in my opinion.

We agree with the reviewer and have added more clarification in the introduction as suggested. We agree this is not an obvious phenomenon and needs more explanation without trying to provide a mechanistic model – as we simply don’t know without experimentation – as the reviewer points out.

Most importantly we strongly agree with the reviewer that there is no experimental validation or functional implications for reservoirs. We agree that the text in the previous version would imply some knowledge of such. We have now toned down all implications that anything is actually known – we are simply pontificating in the discussion and mapping properties to them in the results.

6) Do the peaks associated with reservoir promoters have the same features as all the other peaks in the datasets? For example, do they have the same width? If you consider the distance distribution from annotated TSSs, does that match?

We appreciate the reviewer’s good questions. We had compared the binding profiles of each DNA binding protein between lncRNAs and mRNAs and didn’t observe obvious differences. However, we did not directly compare the width of the profile of DNA binding proteins on reservoirs. To this end, for the DBPs that had sufficient binding in reservoir promoters (>100 peaks), we generated promoter binding profile plots for reservoirs versus non-reservoirs. In many cases, the reservoir profile is wider and less localized to the TSS (notably E4F1, KDM1A, IKZF1, for ex.), and in other cases that do not show uniformly distributed binding there are some distinct deviations such as more upstream binding in the case of HGDF. Based on the reviewers’ suggestion we added this as Supplemental Fig. 7.

Additionally, we compared the individual peak’s widths between reservoirs and non-reservoirs. Here, despite some DBPs with small deviations in the peak width distribution such as an enrichment for smaller peaks in reservoirs for ZBTB33, no systematic difference in peak width was found. Thus, it is not likely that the more diffuse TSS binding profiles seen in reservoirs are a result of a fundamentally different binding mode.

But also do the genes associated with reservoir promoters have "typical" features? In terms of exon size, exon number and/or number of transcripts associated with them.

We really like the reviewer’s suggestions and questions but are concerned by the preponderance of lncRNAs in reservoirs. This alone would skew aspects of “exon count”, splice variation, etc. The most statistically significant feature we see is the bias towards lncRNAs (quite strong). We feel that is the most appropriate distinction of “annotation” features.

Are these genes supported by different systems for the annotation of the human genome: i.e Gencode and Refseq?

We used the Gencode v32 annotation for this analysis and note that all of the reservoir genes are supported at the level of manual annotation or verified loci (which are validated by Gencode’s experimental pipeline (<http://europepmc.org/article/MED/22955982>). However, the reviewer raises a good point about annotation quality. Therefore, we looked at the overlap of the promoter regions derived from Gencode with the gene bodies in the current version of RefSeq (accession: GCF_000001405.39) and found that compared to the overall rate of non-overlapping Gencode promoters (22%), reservoirs do have a higher rate of not having a matched gene in RefSeq. However, the majority of reservoirs do have an overlap with a RefSeq gene(s) (64%). While this may indicate variable expression of these genes, thus motivating looking at the expression resulting from these promoters across cell types, we point out that the surprising part of these regions isn’t the merely the lack of transcription, but rather the sheer variety of binding events in a 6kb window coupled with the lack of transcription.

We wanted to further investigate a more stringent set of reservoir promoters based on this annotation question somewhat implied by the reviewer’s suggestion. In order to dispense with annotation accuracy over the exons of the gene as a potential confounding issue, we wanted to look at whether there were any reads within these reservoir promoters. Specifically, we quantitated RNA sequencing in the same window as reservoirs were defined (3Kb upstream and downstream of each promoter TSS). We found that 356 reservoirs did not have expression in this promoter window. While the remaining reservoirs did have expression in the 6kb window we cannot determine if the reservoir is regulating this expression without experiments to test this. We now include this analysis in the main text and a list of conservative reservoirs in Supplemental Table 2.

If you take large datasets of gene expression across different tissues are these genes expressed? I believe that this type of analyses could help you to understand if some of the patterns that you observe could be associated with biases in reads mapping and/or the annotation of the genome

This is a great question/suggestion and one we are exploring in this year’s class. We are taking a slightly different approach of re-performing these analyses in a different cell type (using our single-cell state approach – where all the data is matched in the same cell context). We will be asking many questions that we feel are beyond the scope of this study. Specifically, we want to ask: Are reservoirs cell type specific? Are reservoirs still present in other cell types and turn on and off expression? What are the features that distinguish reservoirs from super-enhancers in different cell types?

Overall, we agree with the reviewer on how informative their suggestion would be. We are simply taking a more laborious deep-dive into a single “cell state” approach and once we have done this for a few cell types with all the data available for that cell type – then we will know if they are ubiquitous or cell specific.

Additionally, this point made us realize the potential issue of variability across K562 RNA-seq datasets. Since our initial definition of reservoirs relied on only one total RNA-seq experiment, we wanted to be sure more generally that in K562 cells there is no expression in these regions. Therefore, for the conservative list of 356 reservoirs which had no expression in the promoter windows (as described above), we included K562 poly-A selected RNA-seq from three different labs – for a total of four RNA-seq experiments. For this conservative list we required that the median TPM in these promoter regions across all four experiments be less than 0.01. We now include this additional criterion in our conservative reservoir definition for future work on the functional aspects of reservoirs.

7) Although maybe not the main point of the paper. To confirm your findings and demonstrate that "reservoir promoters" are real, you should demonstrate that you observe the same patterns (but ideally at also at different loci) in at least another cell line. If additional analyses cannot be performed, please tone down and discuss this in more details in the discussion.

Indeed, this is the goal of this year’s class across ENCODE lines with enough data (HEPG2, HEK293) to compare to K562. We will indeed tone down and suggest that we can’t say much without comparing to other cell lines. We completely agree but feel this requires a thorough examination (as described in point 6) beyond the scope of this study.

8) In general, the resolution of the figures is low. I do not know if this is due to some problem/flaws in the conversion of the figures in pdf format in the PLOS-ONE online submission system. But please try to provide figures of higher quality. Some text is hardly intelligible. Maybe you should attach a pre-compiled pdf with all the figures in the revision

We will look into this as all images are vector based and should be at clear resolution. Ironically, this was part of the class to teach them to print .eps or .pdf for figure making. We assure the reviewer these will be of highest quality vector graphics. We are not sure what happened here.

9) Having a figure (even a supplementary) and/or a section to show- in general- which type of genomic features and in which proportion are associated with ChIP-seq peaks (gene bodies, promoters, enhancers, intergenic) could be useful. Also, for lines 165-180? maybe you could try to see if there are some patterns and if you can classify your DNA binding proteins based on the type of occupancy that they have on the genome

Yes, this a great suggestion. We previously looked only at peaks overlapping promoters and gene bodies (Fig 2) and at preferences for lncRNA vs protein coding promoters (Fig 3D). We also used UMAP dimensionality reduction to address the reviewer’s suggestion of classifying DBPs based on their binding positions. We found that there was no relationship between type of DNA binding domain, nor TF status. However, based on the reviewer’s suggestion we looked at more features. Specifically, we plotted the HOMER peak annotations which includes intron, exon, TTS, intergenic, and promoter features. We’ve added this as Supplemental Figure 3 and the underlying data in Supplemental Table 3. We added observations regarding this new plot in the text around line 300 in the discussion of promoter binding where we felt it was most appropriate. Overall, classifying these DBPs based on their feature binding would be fuzzy at best, consistent with our UMAP analysis.

10) Since the majority of reservoir promoters are associated with non-coding genes, does the correlation of number of binding events with expression level improve if you exclude these from your analyses?

We see the reviewers point, however the absolute number of lncRNA reservoirs is quite small (981 or roughly 6% of lncRNAs). Thus, this would not likely have much of a global influence and or the maximum effect size would be very small. Our binding versus expression trend is highly consistent with several previous studies that demonstrate lncRNA expression is correlated with binding events but always an order of magnitude lower in expression than mRNAs. These previous studies would have also included non-expressed promoters to fully represent the spectrum of expression levels.

Thus, removing the 6% of lncRNA reservoirs may slightly shift the correlation, but would not affect the overall trend observed here and other studies. Thus, we prefer to be consistent with other studies that globally compare all mRNA and lncRNA promoters (PMID: 27927715, PMID: 30683753, PMID: 21890647, PMID: 22955988).

Anyway, and partially off the records. I teach R and bioinformatics too. I have to say that my class would hardly be capable to such and impressive amount of work. I am impressed! So, keep going with the good work. You are almost there

We can’t tell you how much we appreciate this comment and review – we are very grateful that was informative and inspirational to students!!
